# Apigenin Isolated from the Medicinal Plant *Elsholtzia rugulosa* Prevents β-Amyloid 25–35-Induces Toxicity in Rat Cerebral Microvascular Endothelial Cells

**DOI:** 10.3390/molecules16054005

**Published:** 2011-05-13

**Authors:** Le Zhao, Lin Hou, Huijun Sun, Xin Yan, Xifeng Sun, Jianguang Li, Yong Bian, Yu Chu, Qingshan Liu

**Affiliations:** 1 Beijing Entry-Exit Inspection and Quarantine Bureau, Beijing 100026, China; 2 Shandong University Affiliated Jinan Central Hospital, Jinan 250013, China; 3 Key Lab of Ministry of Education, National Research Center for Minority Medicine, Minzu University of China, Beijing 100081, China

**Keywords:** Alzheimer’s disease, amyloid-β peptide, apigenin, *Elsholtzia rugulosa*, microvascular endothelial cells

## Abstract

Endothelial cells of cerebral capillaries forming the blood-brain barrier play an important role in the pathogenesis and therapy of Alzheimer’s disease. Amyloid-β peptides are key pathological elements in the development of this disease. Apigenin (4’,5,7-tetrahydroxyflavone) is a plant flavonoid and pharmacologically active agent that can be isolated from several plant species. In the present study, effects of apigenin obtained from the medicinal plant *Elsholtzia rugulosa* (*Labiatae*) on primary cultured rat cerebral microvascular endothelial cells (CMECs) mediated by amyloid-β peptide 25–35 (Aβ_25–35_) were examined. Aβ_25–35_ showed toxic effects on CMECs, involving reduction of cell viability, release of lactate dehydrogenase (LDH), increase of nuclear condensation, over-production of intracellular reactive oxygen species (ROS), decrease of superoxide dismutase (SOD) activity, and breakage of the barrier integrity and function. Based on this model, we demonstrated that apigenin from the medicinal plant *Elsholtzia rugulosa* protected cultured rat CMECs by increasing cell viability, reducing LDH release, relieving nuclear condensation, alleviating intracellular ROS generation, increasing SOD activity, and strengthening the barrier integrity through the preservation of transendothelial electrical resistance, permeability property and characteristic enzymatic activity after being exposed to Aβ_25–35_. In conclusion, apigenin isolated from *Elsholtzia rugulosa* has the ability to protect rat CMECs against Aβ_25–35_-induced toxicity.

## 1. Introduction

Cerebral microvascular endothelial cells (CMECs), contributing to form blood-brain barrier (BBB), are critical for creating and maintaining brain homeostasis, and are also the primary targets in cerebral ischemic injury and neurodegenerative conditions [1,2]. Nowadays, many researches suggest that fibrillar Aβ accumulates at sites in the cerebrovasculature, particularly around arterioles and capillaries of the cerebral cortex and leptomeninges [3,4], resulting in cerebral amyloid angiopathy (CAA). Some extent of CAA has been identified more than 80% of Alzheimer’s disease (AD) patients [5], and the severity of microvascular CAA correlates with markers of progression in this disease [6].

Cerebrovascular Aβ deposition plays a role in AD progression. Morphological abnormalities in the cerebral microvasculature in AD include atrophy and irregularities of arterioles and capillaries, swelling and increased number of pinocytic vesicles in endothelial cells, disruption of the basement membrane, reduced total microvascular density and occasional swelling of astrocytic end feet [5,7]. The morphological changes are accompanied by functional alterations, and microvascular segments representing CAA or surrounded by amyloid plaques show increased permeability to endogenous albumin [8]. Moreover, at sub-toxic concentrations, Aβ reduces endothelial antioxidant efficacy [5,6,7], stimulates secretion of inflammatory cytokines [9], up-regulates vascular cell adhesion molecules and increases transendothelial migration [10]. Accordingly, the barrier integrity and function of BBB is seriously affected, showing the disruption of tight junctions, the increased endothelial monolayer permeability [11,12], the altered transport of molecules at the BBB, and the imbalance of characteristic enzymatic activity, such as γ-glutamyl transpeptidase (γ-GT) and alkaline phosphatase (ALP) [13]. Thus, current understanding places the BBB at the epicenter of AD pathophysiology and recognizes brain endothelial cells as new therapeutical targets in AD [14,15].

**Figure 1:**
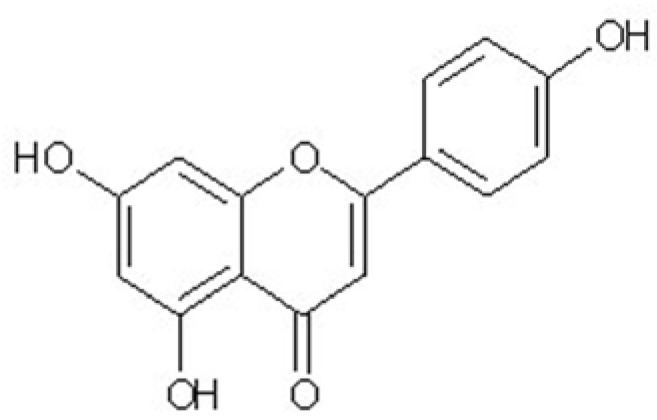
Structure of apigenin.

*Elsholtzia rugulosa* (*Labiatae*) is widely distributed in the Sichuan, Yunnan and Guizhou provinces of China and is well-known locally as a herbal tea, medicinal herb and honey plant [16]. In these regions, the title plant is used by in the treatment of colds, headaches, pharyngitis, coughs and fevers [17]. Several flavonoids, maltol glycosides and cyanogenic glycosides have been isolated from *Elsholtzia* *rugulosa*. Flavonoids such as apigenin (Figure 1) are ubiquitous plant secondary metabolites and have a variety of biological effects, including anticarcinogenic, anti-inflammatory, and free radical-scavenging activities in a variety of *in vitro* systems, or when administered *in vivo* by injection [18,19,20,21,22,23]. It is also noted that apigenin has vasorelaxing, anti-platelet and anti-oxidant properties, which could actually reduce the risk of coronary heart disease and improve endothelial function [24,25,26,27]. Even though some evidence suggests that apigenin has potential effects on endothelial cells, no preexisting study has been reported the protective activity of apigenin isolated from the plant *Elsholtzia rugulosa* on cerebral microvascular endothelial cells mediated by amyloid-β peptide. Thus, as a part of our ongoing screening program to evaluate the protective potential of natural compounds, we investigated the in *vitro* protective activity of *Elsholtzia rugulosa* through activity-guided fractionation. Subsequently, the effects of apigenin isolated from *Elsholtzia rugulosa* were evaluated on Aβ-induced toxicity in the rat primary cultured cerebral microvascular endothelial cells.

## 2. Results and Discussion

In this study, our findings indicate that apigenin isolated from *Elsholtzia rugulosa* had the ability to protect rat CMECs against Aβ_25–35_-induced toxicity. In these effects, apigenin increased cell viability, reduced LDH release, relieved nuclear condensation, alleviated intracellular ROS generation, increased SOD activity, and strengthened the barrier integrity through the preservation of transendothelial electrical resistance, permeability property and characteristic enzymatic activity.

As the BBB protects neurons from xenobiotics and regulates the level of neuroactive mediators [14,28], the maintenance of a functional BBB is essential for the treatment of neurological disorders such as cerebrovascular stroke and neurodegenerative disease. Experimental data support the direct toxicity of Aβ on cultured endothelial cells. Treatments with Aβ peptides results in decreased viability in cultured brain endothelial cells [29,30]. Monolayer integrity of cerebral endothelial cells is also affected by Aβ peptides. In good agreement with this notion, we observed the Aβ_25–35_-induced toxicity on cultured rat CMECs, involving the reduction of cell viability, the release of lactate dehydrogenase (LDH), the nuclear morphology, the overproduction of intracellular reactive oxygen species (ROS), the decrease of superoxide dismutase (SOD) activity, and the breakage of the barrier integrity and function.

Apigenin, as a plant flavonoid, is an active oriental medicine ingredient that has been isolated from several plant species and used to cure diseases such as inflammation, allergy and cancer. However, no report has been issued on the protective effects against Aβ-induced toxicity of apigenin isolated from the medicinal plant *Elsholtzia rugulosa* (*Labiatae*) on microvascular endothelial cells or on the mode of action of its active constitutes. In the present study, we speculated that apigenin may play an essential role on CMECs. Cultured rat CMECs were exposed to aggregated Aβ_25–35_ for 24 h, and cell viability, LDH release, nuclear morphology, intracellular ROS level and SOD activity, microvascular endothelial barrier function were detected combined with the action of apigenin.

### 2.1. Apigenin Protected CMECs from Aβ_25–35_-Induced Cytotoxicity

In the present study, apigenin isolated from *Elsholtzia rugulosa* was investigated for protective effects on cerebral microvescular endothelial cells against Aβ_25–35_-mediated toxicity. Firstly, apigenin at different concentrations were incubated with CMECs for 24 h, and they did not show any toxic effects in the CMECs (data not shown here). Further, to evaluate the potential effects of apigenin, we treated the CMECs with apigenin (0.1 μM, 1.0 μM and 10.0 μM) combined with Aβ_25–35_ for 24 h, and further analysis was detected.

The direct protective effects of apigenin on CMECs against Aβ_25–35_-induced toxicity were examined firstly in two cytotoxicity assays. As shown in Table 1, in the MTS assay, the absorbance of CMECs being exposed to Aβ_25–35_ was significantly reduced (*P* < 0.001). Treatment with apigenin attenuated Aβ_25–35_-induced toxicity, and the absorbance of CMECs increased at 0.1 μM, 1.0 μM, and 10 μM in a dose-dependent manner (*P* < 0.01, *P* < 0.001). A similar effect of apigenin was seen in the LDH release assay. 100 μM Aβ_25–35_ produced significant enzyme leakage from CMECs (*P* < 0.001, Table 1), and the fluorescent intensity based on LDH release treated with apigenin significantly decreased at 0.1 μM, 1.0 μM, and 10.0 μM in a dose-dependent manner.

**Table 1:** Effects of apigenin on cytotoxicity in CMECs against Aβ_25–35_-induced toxicity.

Group	Concentration/μmol∙L	Viability/OD490	LDH/Fluorescence
Control	─	1.00 ± 0.08	905.33 ± 60.75
Aβ_25–35_	100	0.57 ± 0.08 ***	1796.17 ± 152.29 ***
Aβ_25__+35_ + Apigenin	0.1	0.69 ± 0.03 ▲▲	1594.17 ± 83.39 ▲
Aβ_25__+35_ + Apigenin	1.0	0.74 ± 0.03 ▲▲▲	1475.17 ± 111.82 ▲▲
Aβ_25__+35_ + Apigenin	10.0	0.84 ± 0.03 ▲▲▲	1374.17 ± 131.10 ▲▲▲

n = 6, *** *P* < 0.001 *vs* control, ▲ *P* < 0.05, ▲▲ *P* < 0.01, ▲▲▲ *P* < 0.001 *vs*. Aβ_25–35_.

**Figure 2:**
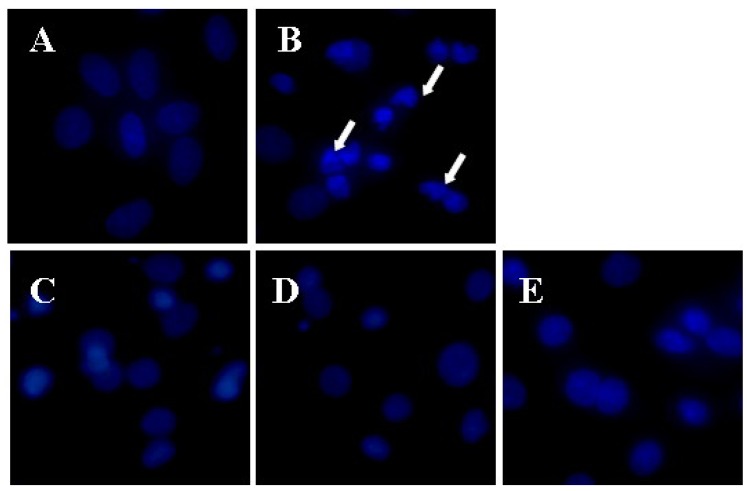
Cytoprotective effects of apigenin by Hoechst 33342 staining against Aβ_25–35_-induced toxicity. Cells were stained with DNA-binding fluorescent dye Hoechst 33342. Nuclear condensation and/or fragmentation are indicative of apoptosis (as indicated by arrows). Images present treatment with (**A**) control; (**B**) Aβ_25–35_; (**C**) apigenin at 0.1; (**D**) 1.0; and (**E**) 10.0 μM and combined with Aβ_25–35_ (×400).

In order to identify whether Aβ_25–35_-induced CMECs cell death through apoptosis and the protective effects of apigenin, DNA staining with Hoechst 33342 was used to evaluate nuclear morphological condensation. As shown in Figure 2, control cells appeared as circles or elliptical shapes where no condensation of the nucleus was observed. In contrast, bright condensed dots or fragmented nuclei (as indicated by arrows) were clearly identified when treated with Aβ_25–35_. The Aβ_25–35_-induced nuclear condensation or fragmentation was significantly attenuated by treatment with apigenin. Hence, Aβ_25–35_-induced CMECs cell death is mainly through apoptosis, and the cytoprotective effects of apigenin were also confirmed by Hoechst 33342 staining by the inhibition of cell apoptosis.

### 2.2. Apigenin Regulated the Redox Imbalance of CMECs Against Aβ_25–35_-Induced Toxicity

Next, intracellular ROS levels and SOD activity were then analyzed. Increased ROS causes deterioration of the cerebral defense system and BBB disruption [31], while SOD exerts protection by scavenging free radicals. In the present study, CMECs in the Aβ_25–35_ group displayed intense fluorescent intensity after being stained with DCFH-DA, which illustrated a remarkable increase of intracellular ROS accumulation resulting from the Aβ_25–35_ exposure (*P* < 0.001, Table 2). Similarly, SOD activity exhibited significantly lower levels in the Aβ_25–35_ group (*P* < 0.001, Table 2). The present data showed that intracellular ROS level and SOD activity were consistently alleviated by the treatment with apigenin (*P* < 0.05, *P* < 0.01, Table 2), indicating apigenin provides sufficient and persistent antioxidant efficacy against Aβ_25–35_-induced toxicity.

**Table 2:** Effects of apigenin on intracellular ROS level and SOD activity against Aβ_25–35_-induced toxicity.

Group	Concentration/μmol∙L	Relative ROS content /Fluorescent intensity	SOD Inhibition rate/%
Control	─	74.74 ± 10.53	33.96 ± 7.73
Aβ_25–35_	100	211.32 ± 22.09 ***	13.60 ± 4.47 **
Aβ_25__+35_ + Apigenin	0.1	175.98 ± 17.10 ▲	20.56 ± 3.23 ▲
Aβ_25__+35_ + Apigenin	1.0	168.72 ± 23.39 ▲	22.82± 3.51 ▲
Aβ_25__+35_ + Apigenin	10.0	157.02 ± 14.54 ▲▲	26.74 ± 3.84 ▲▲

n = 4, *** *P* < 0.001, ** *P* < 0.01 *vs* control, ▲ *P* < 0.05, ▲▲ *P* < 0.01 *vs*. Aβ_25–35_.

### 2.3. Apigenin Improved Barrier Function of CMECs against Aβ_25–35_-Induced Toxicity

Recent findings point out that brain microvascular endothelial cells, contributing to form the BBB, are indispensable for creating and maintaining the homeostasis of the central nervous system (CNS) [3,5,14]. BBB dysfunction leads to disturbed homeostasis, neuronal dysfunction, and secondary neuronal loss [14,28,30]. It has also become clear that even subtle functional changes at the BBB without morphological alterations can lead to severe and lasting neurological dysfunction [5,7]. Therefore the BBB is increasingly considered as a target of new therapeutical approach in neurodegenerative disorders, especially in AD [14,32]. The restrictive nature of the CMECs forming BBB is due in part to tight junctions (TJ) between adjacent endothelial cells [33]. TJs allow for the regulation of ion flux and paracellular diffusion through the development of high transendothelial electrical resistances (TEER) and tight barrier property [34]. The evaluation in this study for the microvascular cell barrier function were TEER, transendothelial permeability and characteristic enzymatic activity.

**Figure 3:**
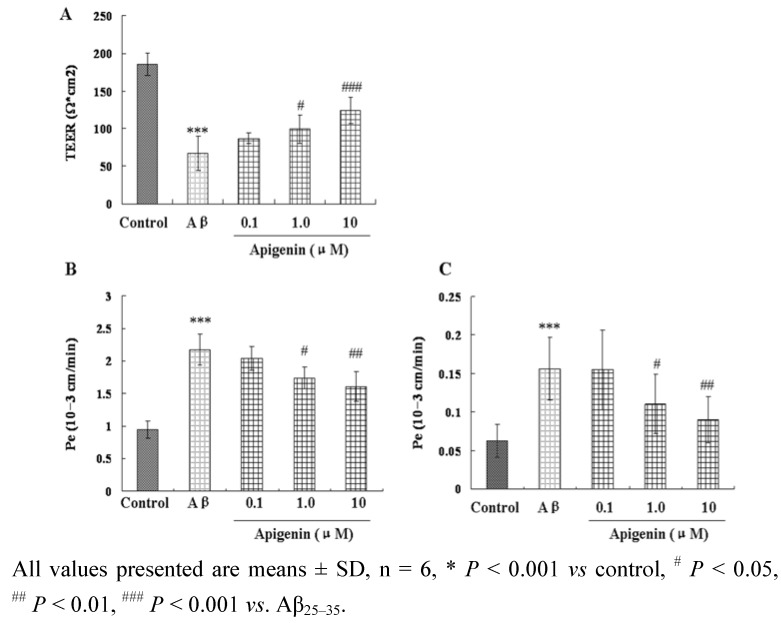
Effects of apigenin on transendothelial electrical resistance (TEER) (A); and transendotheial permeability for sodium fluorescein (B); and FITC labeled albumin (C) in rat CMEC monolayers against Aβ_25–35_-induced toxicity.

TEER is determined by TJ of adjacent brain microvascular endothelial cells, and the integrity of TJ is mainly responsible for the brain endothelial permeability [35]. It is an ideal permeability marker to determine whether TJ is open, and an important indicator to BBB permeability through paracellular pathway. As shown in Figure 3A the value of TEER in the Aβ_25–35_ group was significantly reduced as compared to the control group (*P* < 0.001). The reduction of TEER could be remarkably attenuated by treatment of apigenin at 1.0 μM and 10 μM (*P* < 0.05, *P* < 0.001), indicating that the preservation of monolayer integrity may contribute to its protective effects against Aβ_25–35_-induced damage.

The maintenance of a functional BBB is critical for maintaining low permeability. The permeability studies were performed by permeability markers fluorescein sodium and fluorescein isothiocyanate (FITC)-albumin, to determine the permeability property and paracellular diffusion across confluent CMECs monolayers. The Pe values for permeability marker albumin were lower with about one order of magnitude than the values for fluorescein, a paracellular marker, in agreement with the literature data [36,37]. In the Aβ_25–35_-treated groups, the Pe values were both increased compared with the control group, indicating the serious leakage from the lumen of vessels and the robust increase in the BBB permeability. Apigenin treatments at 1.0 μM and 10 μM attenuated apical-to-basolateral diffusion of fluorescein sodium and FITC-albumin based on the decrease of the Pe values of these two makers (*P* < 0.05, *P* < 0.01, Figure 3B and 3C).

Another property of barrier function we detected was the activity of two enzymes, γ-GT and ALP, both of which are known to be rich on the apical surface of endothelial cells and reliable markers for the BBB [38,39,40,41,42]. A number of stimulatory situations can alter γ-GT and ALP activities, including inflammation [43], oxidative stress [44], and hypoxia [45]. Thus, regulation of enzymatic activity could be another indicator of endothelial malfunction and BBB damage. These two enzymes had the consistent changes with TEER and barrier permeability property, and could be preserved with the treatment of apigenin in a dose-dependent manner at 0.1 μM, 1.0 μM, and 10 μM (*P* < 0.05–0.001, *P* < 0.05–0.001, Table 3). This effect is in agreement with the preservation of monolayer integrity based on TEER and transendotheial permeability, indicating that apigenin isolated from *Elsholtzia rugulosa* might be capable of protecting the barrier integrity and function of endothelial monolayers treated with Aβ_25–35_, and that the exact mode of action at the BBB of this compound could not be elucidated.

**Table 3:** Effects of apigenin on γ-GT and ALP contents against Aβ_25–35_-induced toxicity.

Group	Concentration/μmol∙L	γ-GT/U.mg^−1^ protein	ALP/U.mg^−1^ protein
Control	─	13.47 ± 1.14	13.75 ± 1.49
Aβ_25–35_	100	6.08 ± 1.51 ***	7.96 ± 1.75 ▲▲▲
Aβ_25__+35_ + Apigenin	0.1	8.84 ± 0.94**	10.88 ± 1.71 ▲▲
Aβ_25__+35_ + Apigenin	1.0	10.25 ± 1.26 ***	12.86 ± 2.01 ▲▲
Aβ_25__+35_ + Apigenin	10.0	11.14 ± 0.46 ***	13.73 ± 0.94 ▲▲▲

n = 6, *** *P* < 0.001, ▲▲▲ *P* < 0.001 *vs*. control, ** *P* < 0.05, *** *P* < 0.001, ▲▲ *P* < 0.05, ▲▲▲ *P* < 0.001 *vs*. Aβ_25–35_.

The integrity and barrier function of the brain microvascular endothelial cell monolayer lining the vascular lumen is critical for the maintenance of brain homeostasis. Under physiological conditions, the integrity of the BBB is protected from oxidative stress because the BBB has high levels of antioxidant enzymes [46]. But under pathological conditions, oxidative stress is one of the important mechanisms responsible for the disruption of the BBB. Both endogenous and exogenous ROS may impair endothelial barrier function via mechanisms involving rearrangements of endothelial cytoskeleton and intercellular junctions [47]. Hence, antioxidant therapies scavenging the ROS surplus have long been proposed to protect endothelial barrier function [48]. Administration of apigenin alleviated intracellular ROS generation and increased SOD activity. As one of the natural flavonoids, apigenin showed the potential scavenging activity and antioxidant efficacy by its nature. Additionally, we found that treatment with apigenin was effective in preserving functional integrity of the BBB through the preservation of TEER, permeability property and characteristic enzymatic activity. A relevant effect of apigenin on BBB barrier function by suppressing ROS overproduction might be attributed to the Aβ_25–35_-induced toxicity in mice [49]. Although the precise mechanism by which apigenin exerts its beneficial actions is unclear, antioxidant activity could not be ruled out. Other correlative studies suggest that TJ proteins have been shown to redistribution by the interactions between PKC and Rho/Rho kinase pathways in the structural domain altering with the zipper-like pattern to the linear pattern in the cell-cell boundaries after cAMP treatment [50,51], and that apigenin had the capability to modulate PKC activity through inhibition of cyclic nucleotide phosphodiesterases [52]. Thus, combined with the anti-oxidative property, the capability of apigenin on the BBB regulation might have another explanation.

## 3. Experimental

### 3.1. Reagents

DCFH-DA and Aβ_25–35_ were purchased from Sigma Chemical Co (St. Louis, MO, USA); the latter was dissolved in sterile saline at 1 mM and aggregated by incubation at 37 °C for 7 days before use. Cell Titer96 Aqueous MTS assay kit and CytoTox-ONE Homogeneous Membrane Integrity Assay were purchased from Promega Company (Madison, WI, USA). Dulbecco’s modified Eagle’s medium/Ham 12 (DMEM/F12) and fetal calf serum were purchased from Gibco BRL (Grand Island, NY, USA). The kits for γ-GT and ALP were purchased from Jiancheng Chemical Factory (Nanjing, China). SOD inhibition assay kit and Hoechst 33342 were purchased from Dojindo Laboratory (Kumamoto, Japan). Transwell cell culture inserts (0.4 μm pore size, 12 mm diameter) were purchased from Corning Inc (New York, NY , USA). All other chemicals made in China were analytical grade.

### 3.2. Plant Materials

The whole plant of *Elsholtzia rugulosa* Hemsl. (*Labiatae*) was collected in the Yunnan Province of China, in September 2005, and identified by the Chinese Academy of Medical Sciences. A voucher specimen (ID-14288) was deposited in the Institute of Materia Medica, Chinese Academy of Medical Sciences, China.

### 3.3. Isolation of Apigenin

Whole plant of *Elsholtzia rugulosa* (30 kg) was air-dried, powdered and then refluxed with 95% EtOH (3 × 4,000 mL, 2 h, 1.5 h and 1.5 h, respectively). The combined EtOH solution was filtered and evaporated under reduced pressure to yield 1.675 g of crude extract which was dissolved in 80% EtOH (4,000 mL), and extracted with petroleum ether (60 ~ 90 °C, 3 × 4,000 mL, 2 h each). The petroleum ether (PE) layer was evaporated. Then, evaporation of the aqueous layer under reduced pressure yielded a brown residue which was dissolved in water (8,000 mL), and then extracted with EtOAc (5 × 8,000 mL, 4 h each) to afford an EtOAc extract (140 g). The EtOAc fraction of the plant was subjected to silica gel chromatography with CHCl_3_-MeOH (25:1, R_f_ = 0.78) as the eluent to yield the *Elsholtzia rugulosa* apigenin. The structure of the compound was determined by its physico-chemical and spectral data (LC-MS, 1D and 2D NMR) which were in agreement with those reported in the literature [53]. Sixty (60) mg of apigenin were obtained and the purity of the compound was 96%.

### 3.4. Animals

Male Wistar rats at 3-week-old were obtained from the Institute of Laboratory Animal Sciences, Chinese Academy of Medical Sciences and Peking Union Medical College. All animal experiments were carried out in accordance with institutional guidelines and ethics. 

### 3.5. Cell Culture

Rat cerebral microvascular fragments were isolated and endothelial cells were cultured using methods with modification [13]. Briefly, ten male Wistar rats at 3-week-old were anaesthetized and sacrificed by decapitation. The brains were quickly dissected, and the meninges and choroid plexuses removed. After that, the gray matter of the bilateral cerebral cortex was carefully isolated, and then placed in ice-cold D-Hank’s. The cortices were cut into uniform 1 mm^3^ sections. Sections were then digested in a 0.1% type II collagenase solution for 1 h at 37 °C to separate the microvessels from the other components. Tissue was then centrifuged for 20 min at 4,000 g in 20% bovine serum albumin at 4 °C. The pellet containing crude microvessels was further digested in a second collagenase/dispase solution for 0.5 h at 37 °C. Microvascular fragments were purified using a Percoll gradient at 1,000 g for 10 min. Microvascular fragments were then plated on collagen type IV and fibronectin coated dishes and were cultured in DMEM/F12 containing 10% fetal bovine serum, basic fibroblast growth factor (10 ng/mL), heparin (100 μg/mL), L-glutamine (2 mmol/L), penicillin (100 U/mL) and streptomycin (100 μg/mL). Cultures were incubated at 37 °C in a humid atmosphere of 95% air and 5% CO_2_, and microvascular endothelial cells were grown out rounded the fragments at confluence at about 7 days. When the cultures reached 80% confluency, the purified endothelial cells were passaged by a brief treatment with trypsin (0.05% wt/vol)-EDTA (0.02% wt/vol) solution. The cells were used for experiments from passage 3, containing over 95% CMECs. The CMECs were seeded at 200,000 cells/cm^2^ for 96-well or on the upper side of the polyester membrane of Transwell inserts. The day when the CMECs were plated was defined as Day 0 *in vitro*. On Day 7, the experiments were performed.

Apigenin was firstly dissolved in DMSO at 10 mM and then diluted by DMEM/F12 medium at 10.0 μM, 1.0 μM and 0.1 μM. Apigenin of different concentrations were added at the start of Aβ_25–35_ injury. Apigenin combined with Aβ_25–35_ was incubated for 24 h. CMECs were randomly divided into 5 groups: (1) control group; (2) Aβ_25–35_ group, which was exposed to 100 μM Aβ_25–35_ for 24 h; (3) apigenin at 0.1 μM, (4) 1.0 μM , and (5) 10.0 μM and combined with Aβ_25-35_ for 24 h.

### 3.6. Cell Toxicity Assays

CMECs survival rates were assessed by MTS [3-(4,5-dimethylthiazol-2-yl)-5-(3-carboxymethoxy-phenyl)-2-(4-sulfophenyl)-2H-tetrazolium, inner salt] assay according to the manufacturer’s protocol (Promega, Madison, WI, USA). For MTS assay, the CMECs were cultured in 96-well plates. After treatments the cells were incubated with 20 μL MTS solution for 2 h in CO_2_ incubator, and then determined by measuring absorbance at 490 nm with a microplate reader (Molecular Devices Corp., Sunnyvale, CA, USA).

LDH released from CMECs compromised membranes was determined by the CytoTox-ONE Homogeneous Membrane Integrity Assay (Promega, Madison, WI, USA) according to the manufacturer’s protocol. After treatments 50 μL culture supernatants were transferred to a separate assay plate, leaving behind the cells that were used for ROS detection. 50 μL of CytoTox-ONE Reagent was added to each well, and the contents of the plates were mixed for 30 s. The LDH assay was allowed to proceed at ambient temperature for 10 min prior to addition of 25 μL/well stop solution containing 10% sodium dodecylsulfate. The contents of the wells were mixed by shaking the plates for 10 s prior to measuring resorufin fluorescence (560 nm excitation, 590 nm emission) using a microplate reader (Molecular Devices Corp., Sunnyvale, CA, USA).

According to the Aβ_25-35_ injury and the treatments of different concentrations of apigenin, CMECs were observed under inverted phase contrast microscope to obtain morphological evidence for nuclear condensation/aggregation as previously reported [54]. Cells were plated at a density of 100,000 per well in the 96-well plate. Nuclei were labeled with 5 μg/mL Hoechst 33342 at 37 °C for 10 min after Aβ_25–35_ injury and the treatments of apigenin. Fluorescent images of equal exposure from CMECs were acquired with a digital camera on an Olympus IX71 fluorescent microscope (Olympus Corp., Tokyo, Japan).

### 3.7. Measurements of Intracellular ROS and SOD

Production of reactive oxygen species (ROS) was monitored spectrofluorometrically by the 2’,7’-dihydrodichlorofluorescein diacetate (DCFH-DA) assay with modifications [55]. After treatments culture supernatants were all transferred to a separate assay plate, and DCFH-DA was immediately added to the culture plates at a final concentration of 5 μM and incubated for 40 min at 37 °C in darkness. DCF fluorescence intensity was detected with emission wavelength at 530 nm and excitation wavelength at 485 nm using a microplate reader (Molecular Devices Corp., Sunnyvale, CA, USA). After exposure to Aβ_25–35_, CMECs were collected by scrapping and low-speed centrifugation (1,000 rpm, 10 min). The supernatants were used for SOD. The cells were crushed by sonication (60 W with 0.5 s interval for 15 min). The cell lysate was centrifuged at 10,000 g for 15 min and the supernatant was used to measure the cellular SOD with WST-1 based SOD inhibition assay in a microplate. The solutions in each well were added as described in the manufacturer’s protocol, and the microplate was stirred thoroughly and then incubated at 37 °C for 20 min. The absorbance at 440 nm of the endpoint reaction was measured by using a microplate reader. Percentage inhibition of each sample was calculated by using following equation: {[(*A*_1_ − *A*_3_) − (*A*_S_ − *A*_2_)] / (*A*_1_ − *A*_3_)} × 100, where *A*_1_, *A*_2_, *A*_3_ and *A*_S_ were the absorbance at 440 nm for uninhibited test, blank sample, blank reagent and sample, respectively.

### 3.8. Transendothelial Electrical Resistance (TEER)

TEER was measured using an EVOM resistance meter (World Precision Instruments, Sarasota, FL, USA). The extracellular matrix-treated Transwell inserts were placed in a 12-well plate containing culture medium and should be used to measure the background resistance. The resistance measurements of these blank filters were then subtracted from those of filters with cells. TEER values were expressed as Ω*cm^2^ based on culture inserts.

### 3.9. Transendotheial Permeability for Sodium Fluorescein and FITC Labeled Albumin

The flux of sodium fluorescein (MW: 376Da) and fluorescein isothiocyanate (FITC) labeled albumin (MW: 67 kDa) across endothelial monolayers was determined as previous reports [56]. CMEC culture inserts, following treatment and measurement of TEER, were transferred to 12-well plates containing 1.5 mL Ringer solution (118 mM NaCl, 4.8 mM KCl, 2.5 mM CaCl_2_, 1.2 mM MgSO_4_, 5.5 mM D-glucose, 20 mM Hepes, pH 7.4) in the basolateral compartments. In apical chambers, culture medium was replaced by 500 μL Ringer containing 10 μg/mL sodium fluorescein and 165 μg/mL FITC-BSA. The inserts were transferred at 20, 40, and 60 min to a new well containing Ringer solution. The concentrations of the marker molecules in samples from the upper and lower compartments were determined. FITC and fluorescein levels were measured by a microplate reader (Molecular Devices Corp., Sunnyvale, CA, USA) at emission 488 nm, excitation 535 nm and emission 525 nm, excitation 440 nm, respectively. Flux across cell free inserts was also measured. Transport was expressed as μL of donor (luminal) compartment volume from which the tracer is completely cleared. Transendothelial permeability coefficient (Pe) was calculated as previously described [20,26]. FITC-BSA concentrations in the basolateral chamber, [BSA]basolateral, were determined from a calibration curve of the fluorescence signal, which was linearly dependent upon the BSA concentration.

Permeability, *P*, was calculated according to a linear approximation of Fick’s Law:

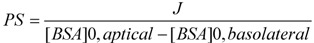
(1)

In formula (1), *S* is the surface area of the transwell insert and *[BSA]0,apical* and *[BSA]0,basolateral* are the initial concentrations of FITC-BSA in the top and bottom chambers, respectively. The flux, *J*, was calculated as the product of the volume of the basolateral chamber times the rate of increase in basolateral FITC-BSA concentration determined from the linear region of [BSA]basolateral *versus* time. Permeability was measured for each experimental group of endothelial monolayers, as well as for membrane supports in the absence of monolayers. Diffusional permeability coefficients (Pe) were then calculated by correction for the permeability of the membrane support in series with the monolayer:

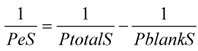
(2)

*PeS* divided by the surface area (1 cm^2^ for Transwell-12) generated the endothelial permeability coefficient (Pe: 10^−3 cm/min).^

### 3.10. γ-Glutamyl Transpeptidase and Alkaline Phosphatase Activity Detection

CMECs were collected by scrapping and low-speed centrifugation (1,000 rpm, 10 min). The supernatants were assayed for γ-glutamyl transpeptidase (γ-GT) and alkaline phosphatase (ALP) activity by using the commercial activity assay kits according to the manufacturer’s protocol. The values were expressed as Umg^-1^ protein as determined by the BCA assay.

### 3.11. Statistical Analysis

Data are expressed as mean ± S.E.M. Each experiment was repeated at least three times. Groups were compared using Student’s two-tailed unpaired *t* test or a one-way ANOVA analysis by employing SPSS 11.0 software, followed by Dunnet’s post-hoc test as appropriate. Statistical significance was set at *P* < 0.05.

## 4. Conclusions

This study deals with the protective effects of apigenin isolated from the plant *Elsholtzia rugulosa* (*Labiatae*) on CMECs against Aβ_25–35_-induced toxicity. We demonstrated that apigenin protected cultured rat CMECs by increasing cell viability, reducing lactate dehydrogenase release, relieving nuclear condensation, alleviating intracellular ROS generation, increasing SOD activity, and strengthening the barrier integrity through the preservation of TEER, permeability property and characteristic enzymatic activity after being exposed to Aβ_25–35_.
